# On the existence and function of galanin receptor heteromers in the central nervous system

**DOI:** 10.3389/fendo.2012.00127

**Published:** 2012-10-26

**Authors:** Kjell Fuxe, Dasiel O. Borroto-Escuela, Wilber Romero-Fernandez, Alexander O. Tarakanov, Feliciano Calvo, Pere Garriga, Mercé Tena, Manuel Narvaez, Carmelo Millón, Concepción Parrado, Francisco Ciruela, Luigi F. Agnati, José A. Narvaez, Zaida Díaz-Cabiale

**Affiliations:** ^1^Department of Neuroscience, Karolinska InstitutetStockholm, Sweden; ^2^St. Petersburg Institute for Informatics and Automation, Russian Academy of SciencesSaint Petersburg, Russia; ^3^Centre de Biotecnologia Molecular, Departament d’Enginyeria Química, Universitat Politécnica de CatalunyaBarcelona, Spain; ^4^Department of Physiology, School of Medicine, University of MálagaMálaga, Spain; ^5^Department of Histology, School of Medicine, University of MálagaMálaga, Spain; ^6^Unitat de Farmacologia, Departament Patologia i Terapéutica Experimental, Universitat de BarcelonaBarcelona, Spain; ^7^Department of Biomedical Sciences, University of Modena and Reggio EmiliaModena, Italy; ^8^Istituto di Ricovero e Cura a Carattere ScientificoLido Venice, Italy

**Keywords:** galanin receptors, heteromers, GPCRs, 5HT1A, NPY receptors, allosteric modulator

## Abstract

Galanin receptor (GalR) subtypes 1–3 linked to central galanin neurons may form heteromers with each other and other types of G protein-coupled receptors in the central nervous system (CNS). These heteromers may be one molecular mechanism for galanin peptides and their N-terminal fragments (gal 1-15) to modulate the function of different types of glia–neuronal networks in the CNS, especially the emotional and the cardiovascular networks. GalR–5-HT1A heteromers likely exist with antagonistic GalR–5-HT1A receptor–receptor interactions in the ascending midbrain raphe 5-HT neuron systems and their target regions. They represent a novel target for antidepressant drugs. Evidence is given for the existence of GalR1–5-HT1A heteromers in cellular models with trans-inhibition of the protomer signaling. A GalR1–GalR2 heteromer is proposed to be a galanin N-terminal fragment preferring receptor (1-15) in the CNS. Furthermore, a GalR1–GalR2–5-HT1A heterotrimer is postulated to explain why only galanin (1-15) but not galanin (1-29) can antagonistically modulate the 5-HT1A receptors in the dorsal hippocampus rich in gal fragment binding sites. The results underline a putative role of different types of GalR–5-HT1A heteroreceptor complexes in depression. GalR antagonists may also have therapeutic actions in depression by blocking the antagonistic GalR–NPYY1 receptor interactions in putative GalR–NPYY1 receptor heteromers in the CNS resulting in increases in NPYY1 transmission and antidepressant effects. In contrast the galanin fragment receptor (a postulated GalR1–GalR2 heteromer) appears to be linked to the NPYY2 receptor enhancing the affinity of the NPYY2 binding sites in a putative GalR1–GalR2–NPYY2 heterotrimer. Finally, putative GalR–α2-adrenoreceptor heteromers with antagonistic receptor–receptor interactions may be a widespread mechanism in the CNS for integration of galanin and noradrenaline signals also of likely relevance for depression.

## INTRODUCTION

Galanin is a neuropeptide (Tatemoto et al., 1983) widely distributed in neurons within the central nervous system (CNS; Jacobowitz et al., 2004). Three Galanin receptor (GalR) subtypes, GalR1–3, have been cloned and belong to the rhodopsin subfamily of G protein-coupled receptor (GPCR; Branchek et al., 2000; Lundstrom et al., 2005; Mitsukawa et al., 2008). GalR1 and GalR2 in particular are found in many regions of the CNS as demonstrated with *in situ* hybridization, radioligand binding, and immunohistochemical studies and have all a high affinity for galanin. GalR1 and GalR3 are coupled to Gi/o leading to inhibition of adenylate cyclase (AC), increases in MAPK activity and opening of G protein-coupled inwardly rectifying K^+^ channels. GalR2 is coupled to Gq/11 and its activation leads to increases in phospholipase C with formation of IP3 increasing intracellular calcium levels and of diacylglycerol (DAG) with the subsequent activation of the protein kinase C. These three GalR subtypes are involved in a number of functions in the CNS modulating neuroendocrine, cardiovascular and mood regulation, pain control, food intake, and seizure threshold (Lundstrom et al., 2005; Mitsukawa et al., 2008). Galanin has also been demonstrated to exert neurotrophic and neuroprotective actions. As early as 1988 (Fuxe et al., 1988b) indications for the existence GalR–5-HT1A receptor–receptor interactions were obtained in rat limbic membranes and in 1994 (Mazarati et al., 1994) in a neuropharmacological analysis results were obtained suggesting interactions of GalRs with glutamate receptors in the dorsal striatum.

This review serves to summarize the indications that GalR subtypes may form heteromers with each other and other types of GPCRs in the CNS as a molecular mechanism to modulate the function of different types of glia–neuronal networks in the CNS.

## RECEPTOR HETEROMERS AND THEIR ALLOSTERIC RECEPTOR–RECEPTOR INTERACTIONS VIA THE RECEPTOR INTERFACE

We began to test the hypothesis of intramembrane receptor–receptor interactions in 1980–1981 in membrane preparations of various CNS regions and found that neuropeptides could modulate the binding characteristics, especially the affinity of the monoamine receptors, in a receptor subtype specific way (Agnati et al., 1980; Fuxe et al., 1981, 1983). Thus, intramembrane receptor–receptor interactions did exist in addition to indirect actions via phosphorylation and changes in membrane potential. However, it took around 10 years before they began to have an impact in the receptor field. But the good news were that the results were in line with earlier findings by Limbird et al. (1975), showing negative cooperativity in beta-adrenergic receptors, which could be explained by the existence of receptor homodimers leading to receptor–receptor interactions. It was also clear that adapter proteins could be involved in mediating the receptor–receptor interactions in brain membranes. A logical consequence for the indications of direct physical interactions between neuropeptide and monoamine receptors, the term heteromerization was introduced to describe a specific interaction between different types of GPCRs (see Zoli et al., 1993; Fuxe et al., 2012a).

Fluorescence resonance energy transfer (FRET) and bioluminescence resonance energy transfer (BRET) methods gave the evidence needed to demonstrate heteromers among class A GPCRs. In FRET two putative proteins can, e.g., bear a “donor” (CFP or GFP2) or an “acceptor” (YFP). If these two proteins interact then the donor and acceptor fluorophores are likely in proximity (10 nm or less) and energy transfer between donor and acceptor can occur after donor excitation by demonstration of YFP emission. In BRET two putative proteins bear a “donor” (Rluc) or an “acceptor” fluorophore (YFP or GFP2). If these proteins are in proximity they do interact. Then, a donor–acceptor energy transfer can occur after Rluc substrate (h-coelenterazine) oxidation. The bioluminescence formed can activate YFP and YFP emission develops (Fernández-Dueñas et al., 2012; Fuxe et al., 2012a).

More recently, evidence has been presented by means of *in situ* proximity ligation assay (*in situ* PLA) that in HEK293 cells D_2L_R can form heteromers with D_4.7_R and especially with D_4.2_R and D_4.4_R (Borroto-Escuela et al., 2011). *In situ* PLA have the potential to enable a fuller understanding of GPCR receptor–receptor and could be highly suited to investigate GPCR heteromers in tissue, providing new insights in basic biological mechanisms, heteroreceptor levels and their locations, e.g., in the brain (Borroto-Escuela et al., 2012).

It is clear that allosteric mechanisms make possible the integrative activity taking place intramolecularly in monomers and intermolecularly in homomers and heteromers (see Fuxe et al., 2010b). Allostery is a mode of long distance communication between distal sites in proteins. There may or may not exist a conformational change at the binding site by the allosteric communication. The conformational change is only one of the possible scenarios. We have preferred pathways which vary with given conditions, through which the strain energy is released from the allosteric site following a perturbation event which can pass over the receptor interface into the other protomer of the receptor homomer or heteromer (see Fuxe et al., 2010a). Kenakin (2008) rightly regards “Seven TM receptors as Nature’s prototype allosteric protein: de-emphasizing the geography of binding”. The allosteric receptor–receptor interactions induced by activation of one receptor protomer can influence the recognition, G protein-coupling and signaling and trafficking of the other protomers in the receptor heteromer.

A high energy strength double arginine-phosphate electrostatic interaction has been found in the A_2A_R–D_2_R heteromer by Ciruela et al. (2004) and Woods et al. (2005) which possess a covalent-like stability as demonstrated with mass spectrometry in combination with collision-induced dissociation experiments and confirmed by pull-down techniques. Based on a mathematical approach, Tarakanov and Fuxe (2010) have deduced, based on 48 pairs of receptors that form or not form heterodimers, a set of triplet amino acid homologies that may importantly participate in receptor–receptor interactions with an origin from integrin triplets of marine sponges and toll-like receptor triplets (Tarakanov et al., 2012a,b). We show how such triplets of amino acid residues and their “teams” may be utilized to construct a kind of code that determines (and/or predicts) which receptors should or should not form heterodimers. We propose a “guide-and clasp” manner for receptor–receptor interactions where “adhesive guides” may be the triplet homologies. Ciruela et al. (2004) showed an amazing stability of epitope–epitope electrostatic interaction based on arginine phosphate. Thus, we should note that two main players contain R (Arg): IDR and AAR. Others have distinct relationships to negatively and positively charged amino acids.

**FIGURE 1:**
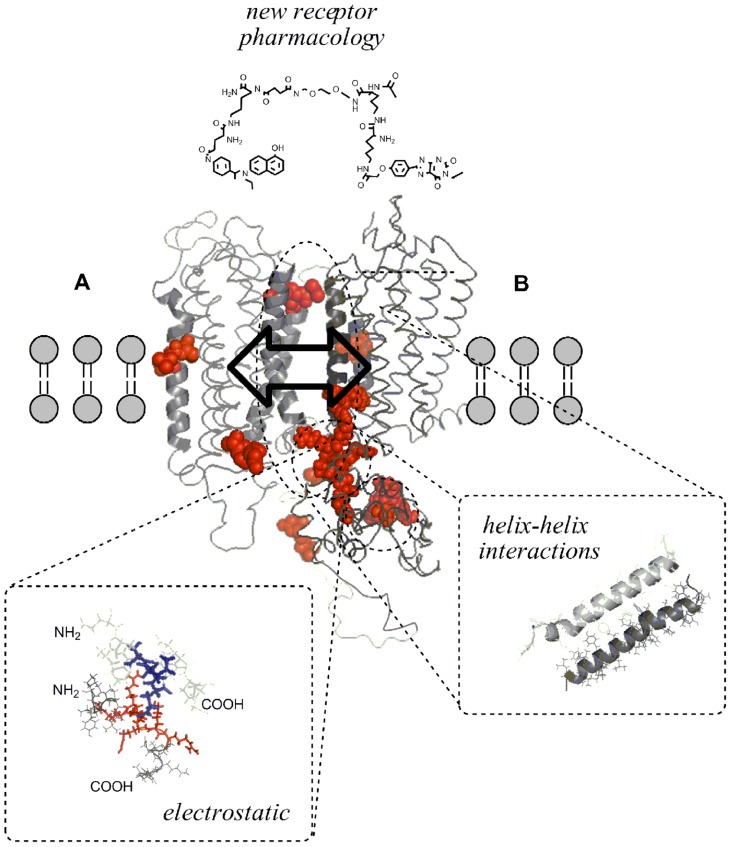
**Intermolecular allosteric mechanisms of the GPCR homo/heteromer and their receptor interface making possible molecular integration of transmitter signals**. Three-dimensional molecular models of the seven TM regions of two putative GPCR (A and B) were built by means of the homology modeling program Accelrys Discovery Studio 2.5 (San Diego, CA, USA) to show that dimerization of GPCR can result from either covalent and non-covalent unions between receptor protomers. (*Helix–helix interaction*) Seen from a lateral view, representation of the A(TM-IV/)–B(TM-V) interaction in the A–B receptor heterodimer is shown. (*Electrostatic interactions*) Illustration of positively charged arginine-rich epitope (red) in the N-terminal part of the third intracellular loop of receptor A electrostatically interacting with the negatively charged (blue) C-terminal epitopes of the receptor B. These electrostatic interactions may represent important hot spots in the receptor interface of some receptor heteromers like A_2A_R–D_2_R, A_2A_R–D_3_R, and A_2A_R–D_4_R heteromers. Allosteric mechanisms make possible the integrative activity taking place intramolecularly in monomers or intermolecularly in homo/heteromers (arrow with two directions in the interface). Intermolecular allosteric mechanisms take place through the formation of different types of receptor homo/heteromers and receptor/protein complexes which can change the function of an individual receptor protomer present in a homomer or heteromer. One example of the novel pharmacology created by heteromers is the use of heterobivalent ligands containing an A pharmacophore and a B pharmacophore linked through a spacer of variable length which may function as useful molecular probes for targeting the A–B receptor heteromer and in this way counteracting or enhancing the receptor–receptor interactions in these heteromers. Such compounds may have a potential for use in pharmacotherapy of CNS diseases.

The receptor heteromers due to the receptor–receptor interactions (RRI) represent a new target for drugs since they allow *inter alia* a higher selectivity in drug actions. Thus, in principle, it is possible to have not simply drugs acting as agonists, or partial agonists or antagonists on a receptor A that is assembled in a heteromer. Rather it is also possible to modulate receptor A through an action on receptor B which belongs to the same heteromer and interacts, via allosteric RRI, with receptor A (**Figure 1**). Against this background, a new field for pharmacology can be considered. Let us list the possible new opportunities to be investigated in a very simple case that of A–B-receptor heterodimers with the aim to modulate the recognition/decoding processes of receptor A. Due to the RRI in the dimer it could be possible to use: (1) receptor B-agonists, (2) receptor B-partial agonists, (3) receptor B-antagonists, and (4) receptor B allosteric modulators. These treatments could change the recognition/decoding process of:

(a) The complex endogenous ligands/receptor A.
(b) The complexes receptor A-agonist/receptor A, receptor A-partial agonist/receptor A, or receptor A-antagonist/receptor A or receptor A allosteric modulator/receptor A.

In the case of a trimer or even higher order oligomers that is of a receptor mosaic (RM) we should consider especially

(a) Topological organization.
(b) Localization and route of the “Allosteric Pathways” inside the RM.
(c) Possible existence of “Check-Points” along the allosteric pathways.

## POTENTIAL EXISTENCE OF GALANINR–5-HT1A HETEROMERS WITH ANTAGONISTIC GalR–5-HT1A RECEPTOR–RECEPTOR INTERACTIONS IN THE ASCENDING MIDBRAIN RAPHE 5-HT NEURON SYSTEMS AND THEIR TARGET REGIONS. A NOVEL TARGET FOR ANTIDEPRESSANT DRUGS

A substantial density of high affinity GalRs was demonstrated in the dorsal raphe by the Jacobowitz group in 1986 (Skofitsch et al., 1986) after having shown the existence of galanin IR cell bodies in the dorsal raphe (DR) after colchicine treatment (Skofitsch and Jacobowitz, 1985). This work inspired the Fuxe group to perform intraventricular (ivt) injections with galanin and evaluate its effects on regional 5-HT levels and metabolism (Fuxe et al., 1988a). In 1986 the coexistence of 5-HT and galanin IR in DR cell bodies was found (Melander et al., 1986) and in 1990 only a proportion of the 5-HT nerve cell bodies in the DR was found to costore galanin and 5-HT IR after colchicine (Fuxe et al., 1990). Intraventricular galanin reduced 5-HT metabolism in ventral limbic cortex, hippocampal formation, and fronto-parietal cortex probably via direct inhibitory actions on DR 5-HT nerve cells reducing their firing rates (Fuxe et al., 1988a). These results for the first time suggested based on the 5-HT hypothesis of depression (see Carlsson et al., 1968) that galanin, via actions on GalRs, mainly GalR in the DR, may contribute to depression by reducing firing in the ascending 5-HT neurons (see also Kehr et al., 2002). Thus, GalR antagonists may represent novel antidepressant drugs.

The same year it was also discovered in limbic membrane preparations that galanin in nanomolar concentrations can reduce the affinity of postjunctional [^3^H]-5HT1A agonist binding sites suggesting that galanin can reduce 5-HT1A recognition and probably signaling in the limbic system (Fuxe et al., 1988b). This indicates that galanin also via such actions can contribute to development of a state of depression, since postjunctional 5-HT1A receptors likely is one of the 5-HT receptors elevating mood upon activation (see Fuxe et al., 1991). This receptor has also been implicated in the pathophysiology of depression based *inter alia* on analysis of brain samples from depressed suicides (Lowther et al., 1997). These results on GalR modulation of 5-HT1A agonist binding sites gave the first indication that brain GalR/5-HT1A heteromers may exist where GalRs antagonize postjunctional 5-HT1A recognition and signaling via intramembrane receptor–receptor interactions (Fuxe et al., 1990, 1991, 2008; Zoli et al., 1993). The antagonistic GalR/5-HT1A receptor interactions in putative receptor heteromers represented a novel integrative mechanism in 5-HT neurotransmission. The role of galanin in the modulation of 5-HT neurotransmission became, however, more complex with the observations that galanin can increase potassium-induced 5-HT release from synaptosomal preparations (Martire et al., 1991). This gave the first indication there may also exist GalRs that increase 5-HT neurotransmission and thus may be beneficial for treatment of depression. In fact, it has recently been proposed that activation of GalR2 can produce antidepressant actions (Lu et al., 2005; see Lundstrom et al., 2005; Mitsukawa et al., 2008). However, the overall inhibitory influence of GalRs was in dominance (see Fuxe et al., 1988a,b). The relevance of antagonistic GalR/5-HT1A interactions for depression were discussed especially in the frame of the 5-HT isoreceptor disbalance hypothesis of depression (see Fuxe et al., 1977, 1991; Ogren et al., 1979) and the galanin-induced reduction of activity in the ascending 5-HT neurons (Fuxe et al., 1991). GalRs counteract the postjunctional mood elevating 5-HT1A signaling while the signaling over other subtypes of 5-HT receptors like the 5-HT2-like receptors which may be blocked by classical antidepressant drugs (Fuxe et al., 1977, 1991; Ogren et al., 1979) were less affected. A behavioral correlate has been obtained to the postjunctional antagonistic GalR/5-HT1A receptor interactions (Razani et al., 2001). The activation of 5-HT1A receptors in membrane preparations and in brain sections was in contrast found to increase the affinity of the GalRs in diencephalic and telencephalic areas (Hedlund et al., 1991a). Thus, reciprocal intramembrane receptor–receptor interactions may exist in putative GalR/5-HT1A heteromers where an activation of 5-HT1A receptors increases GalR recognition and probably GalR signaling. Thus, if the 5-HT1A receptor is regarded as a hub receptor and the GalR as the accessory receptor in this receptor heteromer, it seems possible that the GalR mediates an inhibitory intramembrane feedback mechanism to dampen overactivity in 5-HT1A receptor signaling (Fuxe et al., 1988b, 1991; Hedlund and Fuxe, 1996). However, it is still unknown which GalR subtype protomers are modulated by the agonist-induced activation of the 5-HT1A protomer in the GalR–5-HT1A heteromers. In other cases, however, the enhancement of the GalR signaling may be the major action, allowing the 5-HT1A protomer to bring down firing in the raphe nuclei further due to increased opening of the GalR regulated GIRK channels leading to increased hyperpolarization (Hedlund et al., 1991b).

### GalR–5-HT1A AUTORECEPTOR INTERACTIONS IN THE DORSAL RAPHE 5-HT CELL BODIES/DENDRITES

A time-dependent modulatory action by GalRs exist also on 5-HT1A autoreceptors in the DR nerve cells (Razani et al., 2000). Intraventricular galanin produces first a rapid decrease of the 5-HT1A autoreceptor recognition within 10 min (reduction of affinity) in line with the findings at postjunctional 5-HT1A receptors (Fuxe et al., 1998, 2008). Thus, intramembrane antagonistic GalR/5-HT1A autoreceptor interactions in putative GalR–5-HT1A autoreceptor heteromers can be demonstrated at the soma-dendritic level of the DR 5-HT nerve cells. This will, however, not increase their nerve cell firing since galanin itself causes hyperpolarization (Xu et al., 1998) and it may represent only an intramembrane inhibitory feedback to avoid abnormal inhibition of the DR 5-HT nerve cells.

However, this change in the 5-HT1A autoreceptor is followed after 2 h by a delayed rise in 5-HT1A autoreceptor density probably through reduced internalization of the GalR/5-HT1A receptor heteromers (Razani et al., 2000) and/or a galanin-induced increase in maturation and insertion of these heteromers into the plasma membrane. This was associated with a reduction of galanin mRNA levels and of 5-HT1A mRNA levels in the DR supporting this interpretation. Thus, GalR antagonists may exert antidepressant effects in the DR not only by blockade of GalR signaling but also via blockade of the galanin elicited increase in 5-HT1A autoreceptor density.

A major paper in pointing to a role of raphe GalRs in depression and for the use of GalR antagonists as novel antidepressant drugs was published by Bellido et al. (2002). The discovery was made of an increased density of GalRs in the DR of a genetic rat model of depression based on the use of Flinders Sensitive Line rats. This rise of GalR density was associated with a reduction of galanin IR in the DR and an increase in the immobility time in the forced swimming test. This may represent a primary disturbance contributing to development of human depression by reducing firing in the ascending 5-HT pathways to the limbic system and the diencephalon. These results emphasize the introduction of GalR antagonists targeting the DR in treatment of depression. However, the GalR subtypes appear to have a differential role in depression as demonstrated by Bartfai and colleagues (Bartfai et al., 2004; Lu et al., 2005; Barr et al., 2006). Thus, newly developed non-peptide GalR3 antagonists exert antidepressant like activity in various rodent models of depression (Barr et al., 2006). However, fluoxetine and electroconvulsive shock increase Gal mRNA levels in the DR accompanied by increases in GalR2 receptor binding sites (Lu et al., 2005) giving in contrast an antidepressant potential to GalR2 agonists. In fact, GalR2 may be the GalR involved in releasing 5-HT from synaptosomal preparations (Martire et al., 1991). Based on the available information it therefore seems that general GalR and GalR3 antagonists have antidepressant properties while GalR2 antagonists may reduce mood.

## EVIDENCE FOR THE EXISTENCE OF GalR1–5-HT1A HETEROMERS IN CELLULAR MODELS

Previous work has established homodimerization and internalization of GalR1 in living CHO cells using FRET and time lapse confocal imaging (Wirz et al., 2005). Thus, GalR1 can exist as a dimer in the plasma membrane which may undergo desensitization and internalization upon agonist activation with Gal1-29.

We have examined the possible existence of GalR–5-HT1A heteromers in HEK-293 cells co-transfected with GFP2-tagged 5-HT1A receptor and YFP-tagged GalR1 receptor using a proximity-based FRET assay (Borroto-Escuela et al., 2010). In addition, a novel bioinformatic approach to predict receptor–receptor interface interactions was used (Tarakanov and Fuxe, 2010) together with an analysis of signaling.

Upon co-expression of the 5-HT1A-GFP2 and GalR1-YFP cDNA, a significantly higher FRET signal was observed in comparison to the FRET signal obtained from a mixture of cells individually expressing one of the two receptors. It represented a constitutive heteromers since the observed FRET ratios were unaltered by agonist treatments. In cells co-expressing 5-HT1A-GFP2 and GalR1-YFP the two receptors became clearly co-distributed in the plasma membrane at 48 h. And the specificity of this heteromer is indicated by the observation that expression of GalR2 does not block the FRET signal from developing in the 5-HT1A-GFP2 and GalR1-YFP heteromer. Instead, the FRET signal is increased. The understanding of the mechanism underlying this increase may unravel whether the formation of a GalR1–GalR2–5-HT1A higher-order heteromer (RM) takes place (Borroto-Escuela et al., 2010).

**FIGURE 2:**
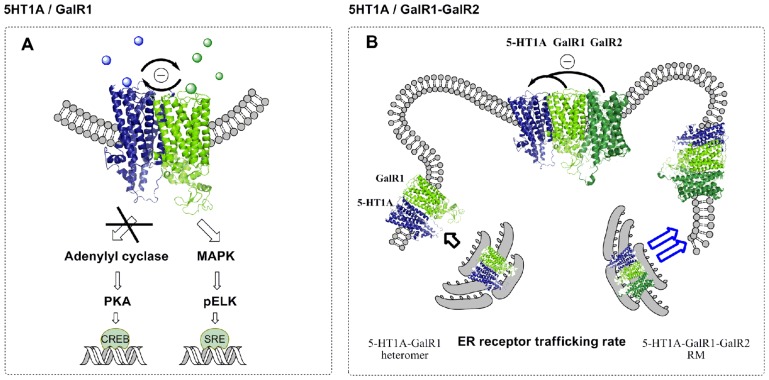
**Recognition, signaling, and trafficking pathways and their antagonistic receptor–receptor interactions in serotonin 5-HT1A and galanin receptor (GalR1) heteromers in HEK293 cells and postulated 5-HT1A–GalR1–GalR2 trimers**. **(A)** Schematic representation of the antagonistic interactions observed after co-treatment with 5-HT1A receptor agonist (8-OH-DPAT) and galanin (peptide 1-29) in co-transfected HEK293 cells. Signaling by either the mitogen-activated protein kinase (MAPK) or adenylyl cyclase (AC) pathways by the 5-HT1A–GalR1 heteromers indicates a trans-inhibition phenomenon through their interacting interface via allosteric mechanisms that block the development of an excessive activation of Gi/o with an exaggerated inhibition of AC or stimulation of MAPK activity. **(B)** A study of the subcellular localization of tagged 5-HT1A and tagged GalR1 in the presence of non-tagged GalR2 showed a co-distribution in the plasma membrane of tagged 5-HT1A and tagged GalR1 already at 36 h in comparison to cells expressing tagged 5-HT1A and tagged GalR1 only. In this case, the tagged 5-HT1A receptor showed a more reticular distribution while tagged GalR1 was mainly found in the plasma membrane. In the presence of GalR2 a co-distribution in the plasma membrane of the tagged 5-HT1A and tagged GalR1 is obtained already at 36 h and is maintained at 48 h. A facilitated interaction of the GalR1–5-HT1A heterodimer into the plasma membrane by expression of GalR2 can be explained based on the postulated receptor mosaic (5HT1A–GalR1–GalR2), where the endoplasmic reticulum (ER) trafficking rate is higher than the trafficking rate shown by the 5-HT1A–GalR1 heteromer. Available findings can be explained by postulating that a GalR1–GalR2 heteromer is a galanin fragment preferring receptor which in the postulated trimer with the 5-HT1A receptor exerts a strong antagonistic receptor–receptor interaction with the 5-HT1A protomer at the level of 5-HT1A recognition.

Using CRE-luciferase and SRE-luciferase reporter assays it was found that signaling by either the MAPK or AC pathways by these heteromers results in a trans-inhibition phenomenon through their interacting interface via allosteric mechanisms that block the development of an excessive activation of Gi/o linked to each of the receptors and an exaggerated inhibition of AC or stimulation of MAPK activity (**Figure 2**; Borroto-Escuela et al., 2010). These receptor heteromers may exist in the ascending raphe 5-HT pathways in view of the demonstration of antagonistic GalR–5-HT1A receptor interactions in the limbic regions and in the raphe reducing the affinity of the 5-HT1A receptors (see above).

Based on a bioinformatics approach, Tarakanov and Fuxe (2010) have deduced a set of triplet homologies that may be responsible for receptor–receptor interactions. This set consists of two non-intersecting subsets: “pro-triplets” and “contra-triplets”. Any pro-triplet appears as a homology in at least one heterodimer but does not appear as a homology in any non-heterodimer. The triplets SNS and LAR may have an important role in the GalR–5-HT1A receptor interface. The locations of the triplet SNS in the same transmembrane domains (TM7) of GalR1 and 5-HT1A were shown. The locations of the triplet LAR were found in the cytoplasmic (intracellular) domains of GalR1 (between TM1 and TM2) and 5-HT1A (between TM5 and TM6). These two triplets may therefore participate in the transmembrane and intracellular components of the interface of the GalR1–5-HT1A heteromer.****

## A GalR1–GalR2 HETEROMER IS PROPOSED TO BE A GALANIN N-TERMINAL FRAGMENT PREFERRING RECEPTOR (1-15) IN THE CNS

**FIGURE 3:**
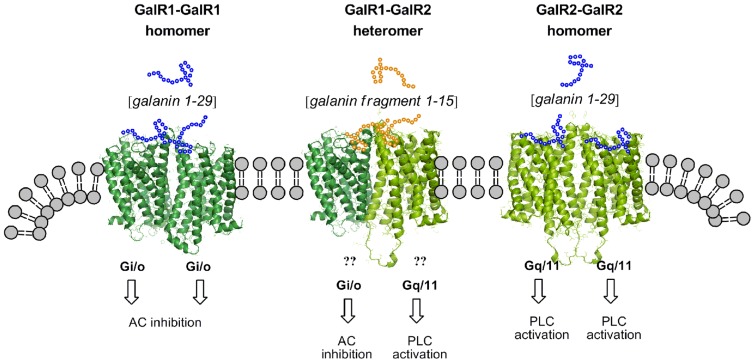
**The unknown signaling of the postulated galanin fragment preferring receptor (GalR1–GalR2 heteromer) is compared with the signaling of the GalR1 and GalR2 homomers**.

The three cloned receptors are known to show a higher affinity for Gal than for galanin N-terminal fragments like Gal (1-15) (Branchek et al., 1998). A substantial further development of this field was the demonstration of specific N-terminal galanin fragment, galanin (1-15) binding sites in the rat brain emphasizing the powerful role of galanin fragments in galanin communication (**Figure 3**), especially in dorsal hippocampus, neocortex, and striatum having few high affinity galanin (1-29) binding sites (Hedlund et al., 1992). Our hypothesis is that these N-terminal Gal fragment preferring sites may be the result of formation of GalR1/GalR2 heteromers leading to conformational changes in their galanin recognition sites converting them into highly specific galanin fragment binding sites with markedly reduced affinity for galanin (1-29) (Fuxe et al., 2008). It is of high interest that gal (1-15) given intraventricularly in the rat has been found to produce marked depression-like behavior in the FST and anxiogenic like effects in the open field (Millon Penuela et al., 2012).

## POSTULATED EXISTENCE OF A GalR1–GalR2–5-HT1A HETEROTRIMER

In agreement with above only galanin (1-15) but not galanin (1-29) can antagonistically modulate the 5-HT1A receptors in the dorsal hippocampus and this effect may be blocked by a known GalR antagonist M35 (Hedlund et al., 1994). Thus, a RM of GalR1–GalR2/-5-HT1A receptors may exist especially in the dorsal hippocampus, neocortex, the striatum, and the raphe where galanin fragments may effectively antagonize postjunctional and autoreceptor 5-HT1A recognition and may function via activation of the postulated GalR1/GalR2 heteromer (**Figure 2**). Also a cross-inhibition of the GalR1–GalR2 heteromer by the 5-HT1A protomer in the trimer should be considered. The GalR1–GalR2–5-HT1A heterotrimer may have a higher trafficking rate from the endoplasmic reticulum to the plasma membrane than the GalR1–5-HT1A heteromer (**Figure 2**; Borroto-Escuela et al., 2010).

Thus, known GalR antagonists should be putative antidepressant drugs also by blocking galanin fragment preferring sites in addition to galanin binding sites increasing postjunctional 5-HT1A mediated 5-HT signaling and the firing of the ascending 5-HT pathways (Hedlund et al., 1994; Fuxe et al., 1998). Therefore, known GalR antagonists may have multiple targets and it would be of high interest to develop an antagonist for treatment of depression that selectively target the GalR1–GalR2 heteromer postulated to be the galanin N-terminal fragment receptor.

Evidence has been presented that N-terminal galanin fragments can more strongly and more potently reduce postjunctional 5-HT1A receptor recognition also in the ventral limbic cortex where also high affinity GalRs exist (Diaz-Cabiale et al., 2000a). These effects were also blocked by a GalRs antagonist. The galanin fragment preferring receptor may again be formed by the heteromerization of GalR1 and GalR2, since they are known to be present here like in the dorsal hippocampus (O’Donnell et al., 1999). The results underline an important role of different types of GalR–5-HT1A heteroreceptor complexes in depression.

## POTENTIAL EXISTENCE OF GalR–NPYY1 RECEPTOR HETEROMERS IN THE NUCLEUS TRACTUS SOLITARIUS (NTS), THE HYPOTHALAMUS AND THE DORSAL RAPHE WITH ANTAGONISTIC GalR–NPYY1 RECEPTOR INTERACTIONS

**FIGURE 4:**
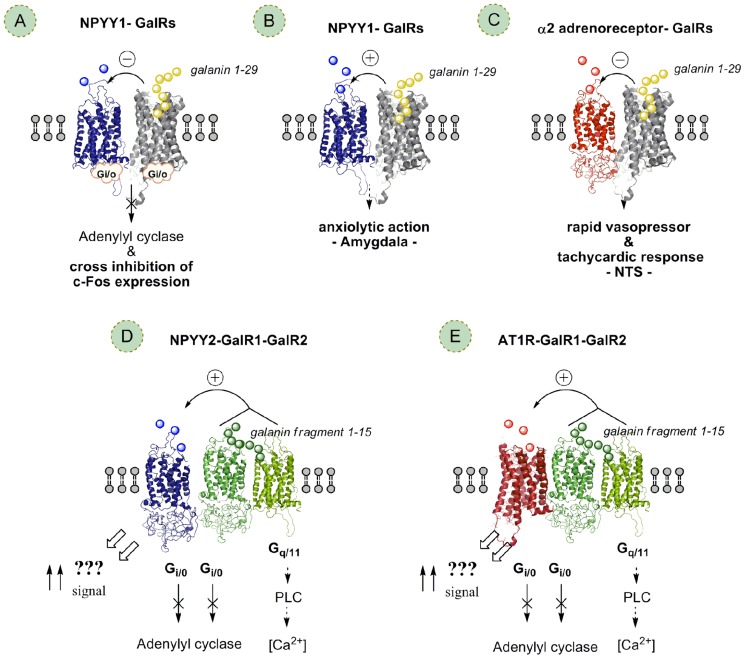
**Different types of postulated GalR heteromers and Gal-fragmentR heteromers (postulated heterotrimers) and their allosteric receptor–receptor interactions**. Galanin receptor (GalR) subtypes1-3 linked to central galanin neurons may form heteromers with each other and other types of GPCRs in the CNS. These heteromers may be one molecular mechanism for galanin peptides and their N-terminal fragments (galanin 1-15) to modulate the function of different types of glia–neuronal networks in the CNS, especially the emotional and the cardiovascular networks. The allosteric receptor–receptor interactions induced constitutively or by activation of one receptor protomer can influence the recognition, G protein coupling, signaling and trafficking of the other protomers in the receptor heteromer. **(A)** NPYY1–GalRs heteromers likely exist with antagonistic NPYY1–GalR receptor interactions involving a reduction of NPYY1 receptor agonist affinity probably taking place in postulated GalR/NPYY1 receptor heteromers in NTS, hypothalamus, and dorsal raphe. GalR antagonists may also have therapeutic actions in depression by blocking the antagonistic GalR–NPYY1 receptor interactions in putative GalR–NPYY1 receptor heteromers in the CNS resulting in increases in NPYY1 transmission and antidepressant effects. **(B)** In contrast, in the amygdala a facilitatory GalR/NPYY1 receptor interaction, likely involving another GalR subtype than in A, is postulated based on the increase of NPYY1 binding induced by GalR activation in this region expected to produce anxiolytic actions. **(C)** Putative GalR–α2-adrenoreceptor heteromers with antagonistic receptor–receptor interactions reducing α2 function may be a widespread mechanism in the CNS for integration of galanin and noradrenaline signals, also of likely relevance for depression. **(D)** Galanin fragment preferring receptor (a postulated GalR1–GalR2 heteromer) appears to be linked to the NPYY2 receptor enhancing the affinity and signaling of the NPYY2 receptor via unknown signaling pathways in a putative GalR1–GalR2–NPYY2 heterotrimer; a reduction of Gi/o signaling may contribute. **(E)** Furthermore, the postulated GalR1–GalR2 heteromer, a postulated galanin N-terminal fragment preferring receptor (1-15), appears to be linked also to the AT1R in a postulated AT1R–GalR1–GalR2 receptor mosaic enhancing the signaling of the AT1R within the NTS via unknown signaling pathways; a reduction of Gi/o signaling may contribute.

In a series of papers by Diaz-Cabiale and colleagues (Diaz-Cabiale et al., 2006, 2011; Parrado et al., 2007) evidence has been obtained for an antagonistic GalR modulation of NPY receptor mechanisms suggesting the existence of GALR–NPYY1 interactions involving a likely reduction of NPYY1 receptor agonist affinity probably taking place in GalR/NPYY1 receptor heteromers (**Figure 4A**) in NTS, hypothalamus, and DR (Diaz-Cabiale et al., 2006, 2011; Parrado et al., 2007). The results open up the possibility that GalR/NPYY1 receptor–receptor interactions in putative heteromers is a frequent phenomenon in CNS with implications for the integrative functions of galanin and NPY in these regions. Thus, the GalR subtype involved has not been determined. This interaction may be based on a GalR/NPYY1 receptor heteromerization where the galanin induced conformational change in the GalR can cause a conformational change in the NPYY1 receptor via the GalR/NPYY1 interface leading to reduced NPYY1 recognition and G protein coupling and thus to reduced NPYY1 receptor signaling.

In the NTS (Diaz-Cabiale et al., 2006) the results suggest the existence of antagonistic GalR–NPYY1 receptor interactions in cardiovascular regions of this nucleus, reducing NPYY1 signaling and thus vasodepressor activity leading to enhanced vasopressor and tachycardic actions of GalR activation in the NTS.

In the hypothalamus (Parrado et al., 2007) evidence was, for the first time, obtained that GalR activation significantly reduced the NPYY1 receptor agonist binding in the hypothalamus without effects on NPYY2 receptor agonist binding. These GalR–NPYY1 receptor interactions have physiological implications since the food intake induced by the NPYY1 receptor agonist is blocked by galanin. The changes observed on c-Fos expression support the hypothesis that GalR activation modulates the response elicited by the NPYY1 agonist.

In the DR we may postulate, on the basis of the galanin-induced decrease in NPYY1 binding, an inhibitory GalR/NPYY1 receptor–receptor interaction that modulates behavioral functions associated with mood and motivation. Behavioral and neurochemical studies support a role of galanin and NPY in mood disorders and GalR1-3 and NPYY1 receptors have been the receptors implicated in depression with GalR subtype specific antagonists and NPYY1 agonists having an antidepressant role (Lu et al., 2005; Lundstrom et al., 2005; Ishida et al., 2007; Jimenez-Vasquez et al., 2007; Fuxe et al., 2008). The decrease of NPYY1 agonist binding induced by galanin and the demonstrated cross-inhibition of c-Fos expression in the dorsal raphe upon GalR and NPYY1 agonist co-activation provides one possible basis for the use of synergistic interactions of GalR subtype specific antagonists and NPYY1 receptor agonists as a strategy for treatment of depression. Thus, GalR antagonists may also have therapeutic actions in depression by blocking the antagonistic GalR–NPYY1 receptor interactions resulting in increases in NPYY1 transmission and antidepressant effects.

In the amygdala (Parrado et al., 2007) we may instead on the basis of the galanin-induced increase in NPYY1 binding postulate a facilitatory GalR/NPYY1 receptor interaction (**Figure 4B**) that could be expected to produce anxiolytic actions. Behavioral studies support a role of galanin and NPY in reducing anxiety in the amygdala and GalRs, NPYY1, and NPYY5 have been the receptors implicated in this effect (see Moller et al., 1999; Heilig, 2004). The increase of NPYY1 binding induced by galanin in this study provides a possible basis for synergistic interactions of GalR agonists and NPYY1 receptor agonists in counteracting anxiety behavior. The differential modulation of NPYY1 binding by galanin in the hypothalamus and in the amygdala could be explained by *inter alia* the involvement of different GalR subtypes in the interaction with the NPYY1 receptor in the hypothalamus and amygdala, respectively. It may also be that the same GalR subtype and NPY receptor subtypes are involved in the two areas but they may be part of different RMs (cluster of multiple receptors; higher order heteromers) leading to altered GalR/NPYY1 interactions in the two areas due to differences in the multiple receptor–receptor interactions (Rimondini et al., 1999) in discrete RMs (Agnati et al., 1982, 2003; Fuxe et al., 2010a,b). Thus, in this way the GalR may reduce NPYY1 signaling in the hypothalamus and increase it in the amygdala.

## POSTULATED EXISTENCE OF A GalR1–GalR2–NPY Y2 HETEROTRIMER IN THE NTS

The presence of specific binding sites for the galanin fragment 1-15 in the nuclei involved in central cardiovascular regulation has been described (Hedlund et al., 1992). In the brainstem a high density of these galanin (1-15) binding sites appears within the NTS, supporting the hypothesis of the existence of a receptor with a higher affinity for the N-terminal fragment than for galanin (Diaz-Cabiale et al., 2005b). We have proposed (see above) that these N-terminal galanin fragment preferring sites may be the result of formation of GalR1/GalR2 heteromers leading to conformational changes in their galanin recognition sites converting them into highly specific galanin fragment binding sites with markedly reduced affinity for galanin (Fuxe et al., 2008). As a matter of fact galanin and N-terminal fragment galanin (1-15) have specific and different roles (hypotensive and vasopressor responses, respectively) in cardiovascular regulation (Diaz-Cabiale et al., 2005b). The N-terminal fragment galanin (1-15) antagonized the cardiovascular effects of galanin (Narvaez et al., 1994) and galanin fragment (1-15) but not galanin decreases baroreceptor reflex sensitivity (Diaz et al., 1996). Galanin and galanin (1-15) also stimulate the expression of c-Fos with different temporal and spatial profiles, especially in the NTS and in the ventrolateral medulla (Marcos et al., 2001). The GalR antagonist M40 is able to block the cardiovascular responses elicited by the N-terminal fragment galanin (1-15) (Narvaez et al., 1994) giving evidence that regular GalR antagonists also can block the galanin fragment preferring receptors (postulated GalR1–GalR2 heteromer).

NPY operates in central cardiovascular regulation through the NPYY1 and Y2 receptor subtypes. Leu31Pro34NPY, a specific NPYY1 receptor agonist, microinjected into the NTS elicits vasodepressor and bradycardic responses (Yang et al., 1993), whereas the injection of the NPY C-terminal fragment (13-36), a specific NPYY2 receptor agonist, leads to vasopressor responses at low doses (Aguirre et al., 1990; Yang et al., 1993). In the study of Diaz-Cabiale et al. (2010) the co-injection of threshold doses of galanin (1-15) and of the NPYY2 agonist resulted in an increase of MAPK of the same magnitude as observed with threshold doses of NPY and galanin (1-15). Galanin (1-15) was also found to specifically increase the NPYY2 agonist binding in the NTS without inducing any effect on NPYY1 agonist binding (Diaz-Cabiale et al., 2010). The increase by galanin (1-15) of the NPYY2 agonist binding may indicate a galanin (1-15)-induced increase of Y2 receptor affinity in the NTS, since the concentration of the NPYY2 agonist used (25 pM) is in the range of the *K*_d_ value, where mainly affinity changes affect the binding level. This interaction may be based on GalR1–GalR2–NPYY2 receptor heteromerization (**Figure 4D**) where the galanin (1-15) induced conformational change in the GalR1–GalR2 heteromer via the interface with NPYY2 can cause a conformational change in the NPYY2 receptor leading to increased NPYY2 recognition and switching of G protein coupling likely to Gq and thus to increased novel NPYY2 receptor signaling producing the vasopressor activity (**Figure 4D**; Diaz-Cabiale et al., 2010). These results illustrate the high impact of the allosteric receptor–receptor interactions in heteromers in the integrative mechanisms responsible for the organization of the cardiovascular responses from the local circuit level of the NTS.

## POSTULATED EXISTENCE OF GalR1–GalR2–AT1 HETEROTRIMER IN THE NTS

Galanin (1-15)/angiotensin II (Ang II) interactions have also been observed in central cardiovascular control (Diaz-Cabiale et al., 2005a). Thus, intracisternal co-injections of threshold doses of Ang II with galanin (1-15) induce a significant vasopressor response that was maintained during the whole recording period, without any significant effect on heart rate. This response was blocked by the AT1 specific antagonist DuP753 (Diaz-Cabiale et al., 2005a). These data suggest the existence of a synergistic interaction between Ang II and galanin (1-15), in which the AT1 and Gal receptor subtypes participate. It may involve allosteric receptor–receptor interactions in a GalR1–GalR2–AT1 heterotrimer (**Figure 4E**) within the NTS and switching of the AT1R to Gq/11 mediated signaling.

## POSTULATED EXISTENCE OF GalR–α2 ADRENORECEPTOR HETEROMERS IN THE CNS

This proposal is *inter alia* based on the effects of galanin on α2-adrenoreceptor activation were evaluated on central cardiovascular regulation in the NTS using also quantitative receptor autoradiography (Diaz-Cabiale et al., 2000b). Central administration of threshold doses of galanin together with an effective vasodepressor dose of the α2-adrenoreceptor agonist clonidine was found to induce a rapid vasopressor and tachycardic response. On the contrary, the co-injection of threshold doses of clonidine and N-terminal galanin fragment (1-15) did not result in any significant cardiovascular change (Diaz-Cabiale et al., 2000b). These functional findings suggest that galanin, but not galanin fragment (1-15), antagonizes α2-adrenoreceptor signaling via a GalR–α2-adrenoreceptor interaction in a postulated heteromer built up of these receptor protomers (**Figure 4C**). The GalR subtypes involved in these heteromers are presently unknown.

Quantitative receptor autoradiography supported this view since galanin decreased the affinity of the α2-adrenoreceptor agonist [^3^H]p-aminoclonidine in the NTS and also increased significantly the density of the α2-adrenoreceptor agonist binding sites. These effects disappeared in presence of the specific GalR antagonist M35, demonstrating that this action is a direct consequence of GalR activation by galanin (Diaz-Cabiale et al., 2000b, 2005b). Galanin also reduced the affinity of the α2-adrenoreceptor agonist [^3^H]p-aminoclonidine in the tel- and diencephalon which was also blocked by the specific receptor antagonist M35 (Diaz-Cabiale et al., 2001). Thus, GalR–α2-adrenoreceptor heteromers with antagonistic receptor–receptor interactions may be a widespread mechanism in the CNS for integration of galanin and noradrenaline (NA) signals.

These antagonistic receptor–receptor interactions participate in the regulation of pre- and postjunctional central NA transmission and many antidepressant drugs exert part of their therapeutic effects by increasing NA transmission via blockade of the NA transporter (Carlsson et al., 1966, 1969). Thus, these antagonistic GalR/α2-adrenoreceptor interactions are of relevance for depression and its treatment.

In the NA cell bodies, dendrites and terminal networks the α2-adrenoreceptor function as NA autoreceptors reducing NA cell firing and NA release from the terminal networks. At this level this interaction will therefore favor increases in NA release and thus NA transmission. At the postjunctional level this antagonistic interaction found all over the tel- and diencephalon will change the balance of the pattern of NA isoreceptor activation and favor the increased activation of beta-adrenergic receptor and α1-adrenoreceptor subtypes. In view of the calming influence of α2-adrenoreceptors at the behavioral level (see Fuxe et al., 2012b) the preferential activation of the other adrenergic receptor subtypes by galanin through the antagonistic GalR/α2-adrenoreceptor interaction may result in increased arousal. It will therefore be interesting to explore how this interaction may be altered in the locus coeruleus and in the limbic networks in models of depression.

## EXISTENCE OF DopamineR–galaninR HETEROMERS IN THE VENTRAL HIPPOCAMPUS

There exists evidence for the existence of D1R–GalR1 and D5R–GalR1 heteromers in cellular models and D1-like receptors upon agonist-induced activation were shown to enhance the GalR1-induced MAPK signaling (Moreno et al., 2011). D1-like receptor antagonists blocked galanin-induced MAPK activation in the ventral hippocampus and in synaptosomes from this region galanin facilitated acetylcholine release upon co-activation of the D1-like receptors. Thus facilitatory allosteric D1-like-GalR interactions in heteromers may exist in control of hippocampal acetylcholine release and electrophysiological experiments in hippocampal slices using field EPSP recordings suggest a modulatory role of the dopamineR–galaninR heteromers in cholinergic neurotransmission (Moreno et al., 2011).

## CONCLUSIONS

GalR subtypes may have a major role in modulating the emotional networks of the brain through heteromerization with 5-HT1A, NPYY1, and α2-adrenoreceptors leading to antagonistic allosteric receptor–receptor interactions producing reductions in 5-HT1A, NPYY1, and α2-adrenoreceptor pre and especially postsynaptic signaling in the central 5-HT and NA neurons. This may be one way in which the activity at certain GalR subtypes and at galanin fragment preferring receptors may contribute to a reduction of mood, which may lead to depression. The GalR heteromers also participate in cardiovascular functions, food intake and regulation of fear and anxiety. The hypothesis is introduced that the galanin fragment preferring receptor is formed by the GalR1–GalR2 heteromer which can mediate the strong depressant actions of Gal 1-15 upon intraventricular injections. Its postulated formation of a trimer with 5-HT1A receptors may represent a novel target for antidepressant drugs.

## Conflict of Interest Statement

The authors declare that the research was conducted in the absence of any commercial or financial relationships that could be construed as a potential conflict of interest.
